# Integration of immunotherapy and radiotherapy in a therapeutic algorithm for locally advanced squamous cell skin cancer

**DOI:** 10.1007/s12032-025-02785-3

**Published:** 2025-06-04

**Authors:** Ioannis M. Koukourakis, Antonios Karpouzis, Konstantinos Filippatos, Panagiotis Mamalis, Despina Kakagia, Alexandra Giatromanolaki, Vassilis Kouloulias, Anna Zygogianni, Michael I. Koukourakis

**Affiliations:** 1 Radiation Oncology Unit, Aretaieion Hospital, School of Medicine, National and Kapodistrian University of Athens (NKUOA), Athens, Greece; 2 Department of Dermatology, Democritus University of Thrace, University Hospital of Alexandroupolis, Alexandroupolis, Greece; 3 Department of Radiotherapy / Oncology, Democritus University of Thrace, University Hospital of Alexandroupolis, 68100 Alexandroupolis, Greece; 4 Department of Plastic Surgery, Democritus University of Thrace, University Hospital of Alexandroupolis, Alexandroupolis, Greece; 5 Department of Pathology, Democritus University of Thrace, University Hospital of Alexandroupolis, Alexandroupolis, Greece; 6 Department of Clinical Radiation Oncology, Attikon Hospital, School of Medicine, National and Kapodistrian University of Athens (NKUOA), Athens, Greece

**Keywords:** Skin cancer, Immunotherapy, Cemiplimab, Radiotherapy, Hypofractionation

## Abstract

Locally advanced squamous cell skin cancer (LA-sqCSC) affects older patients who initially decline medical assistance and patients after multiple surgical interventions for recurrent tumors. Radical radiotherapy (RT) often becomes difficult to apply due to large surface areas, extensive ulceration, and tissue necrosis. Thirty patients with LA-sqCSC were treated according to a therapeutic algorithm involving upfront immunotherapy (IO) with cemiplimab (anti-PD-1 MoAb) and RT. Patients with progressive or stable disease (PgD, SD) or partial/minimal response (PR/MR) received local RT (6 daily fractions of 6 Gy) concurrently with cemiplimab. Complete responders (CR) after IO continued cemiplimab, till progression or development of immune-related adverse events (irAEs) for a maximum of 18 months. irAEs enforced interruption of cemiplimab in 6/30 (20.0%) patients. After the 6th cycle of IO, the CR rates were 50.0%. Nine patients with PR/MR to IO underwent RT. Six of them (66.7%) responded completely. The overall CR rates of patients treated with the proposed algorithm were 70.0%. The 2-year projected locoregional progression-free survival was 68.4% and the disease-specific overall survival was 85.2%. LA-sqCSC can be effectively treated with upfront cemiplimab IO followed by a short course of hypofractionated RT directed to the residual tumor.

**Supplementary Information:**

The online version contains supplementary material available at 10.1007/s12032-025-02785-3.

## Background

Squamous cell skin cancer (sqCSC) is a common malignancy worldwide [1]. Despite the high curability of smaller tumors, locally advanced disease (LA-sqCSC), as a result of multiple recurrences and failure of local therapies, or a consequence of refusal of mainly older patients to seek medical care, is a therapeutic challenge. Very large tumors with extensive ulceration, necrosis, and invasion to adjacent anatomic structures are not candidates for surgery, while application of RT is difficult. Metastatic disease to the lymph nodes and distant organs can also occur, especially in high-grade squamous cell carcinomas, and for these patients, prognosis is poor as chemotherapy has limited efficacy [2].

Following the good results obtained from phase I/II trials and the overall clinical experience, cemiplimab, an anti-PD-1 monoclonal antibody, was approved for the immunotherapy (IO) of patients with advanced inoperable squamous cell skin cancer [3, 4]**.** The reported response rates in the range of 50% in patients with metastatic squamous cell carcinoma, lasting more than 6 months, are certainly encouraging and by far superior to the clinical experience with other systemic therapy [5]**.**

Considering the good results of IO reported for patients with advanced non-melanoma skin epithelial cancer together with the high efficacy of RT, at least in earlier stages [6], the combination of RT with cemiplimab in inoperable locally advanced squamous emerges as a promising therapy to test clinically.

It is suggested that upfront cemiplimab IO could provide substantial tumor responses that could facilitate subsequent radical RT. We tested this hypothesis using a therapeutic algorithm integrating upfront IO followed by RT for the treatment of LA-sqCSC.

## Methods

We report the experience obtained from a prospective study on 30 patients with inoperable, locally far advanced, difficult to treat with RT, squamous cell, or metatypical carcinomas of the skin, recruited in the context of an IO/RT prospective study. Overall, the study protocol aims to build clinical experience on the addition of hypofractionated RT to standard IO in patients with locally advanced (with or without metastasis) epithelial head-neck, lung, pelvic, and skin tumors, who start anti-PD-1 ΙΟ as standard of care (patients with documented resistance to previous chemotherapy, patients unable to receive chemotherapy for any reason, or patients who are eligible to receive first line IO). RT was to be directed to the primary tumor and/or selected metastatic lesions using sub-radical (3 fractions of weekly 8Gy) or radical doses (6 or 8 Gy/week for 6 and 4 fractions, respectively) according to the discretion of physicians who would take into account the individual clinical status and aim of therapy. Radical RT doses were chosen when eradication of the ‘in-field’ disease could provide a chance for cure.

For LA-sqCSC, neoadjuvant IO with the cemiplimab anti-PD1 monoclonal antibody is a proposed therapeutic approach by the National Comprehensive Cancer Network (NCCN) 1.2024 guidelines [7] for very high-risk disease and in patients where curative surgery and RT are not feasible. IO can be administered in an attempt to reduce the tumor burden and, eventually, facilitate RT or even surgery. Given our 20-year excellent experience with treating large skin carcinomas with 6 consecutive fractions of 6-Gy electron irradiation, this regimen was included in the protocol specifically for patients with skin cancer.

The endpoint of the study was to build clinical experience on the feasibility and efficacy of IO/RT regimens for LA-sqCSC, and compare the overall response, locoregional progression-free (LPFS) and overall and disease-specific survival (OS) rates with those reported in clinical trials of IO alone, although such comparisons can be only indicative given eventual differences in the treated populations. The herein clinical complete responses (CRs) could not be directly compared to pathologic responses reported in published studies.

The study has been approved by the local Scientific and Research Ethics Committee (Approval number ES9 04-5-2022). The study was conducted according to the Declaration of Helsinki and the Guidelines for Good Clinical Practice. Moreover, approval for the administration of cemiplimab has been obtained for each patient separately, following application to the National Organization for the Provision of Health Services (EOΠYY). All patients provided written informed consent and approved the use of their laboratory and clinical data for research purposes.

### Recruitment criteria

Inclusion criteria were patients with very large inoperable squamous or metatypical skin carcinomas, difficult to treat with radical RT. Exclusion criteria were Performance Status (PS) > 2, major heart, pulmonary, liver, kidney, active infection, autoimmune disease, HIV infection, organ transplantation, ongoing immunosuppressive therapy, including corticosteroid treatment, and pregnancy.

### Patient and disease characteristics

The median age was 80.5 years and 11/30 patients had poor PS (2 of WHO scale) due to advanced age (not related to the disease). Patients suffered from very large tumors (median size of 7.5 cm), the majority of which displayed extensive ulceration and tissue necrosis (28/30). Patient and disease characteristics are reported in Table 1.

**Table 1:** Patient and disease characteristics

No. of pts	30
Age	
Median	80.5
Range	60–90
Performance status	
0	3
1	16
2	11
Gender	
Female	8
Male	22
Histology	
Squamous	28
Metatypical	2
Location	
Temporal	8
Temporal/nodes	1
Scalp	5
Preauricular	6
Cheek	3
Eyebrow	2
Frontal area	1
Lip	1
Nape	1
Thoracic wall	1
Neck nodes	1
T stage	
T3	24
T4	6
N stage	
N0	29
N2	1
Max dimension (cm)	
Median	7.5
Range	4–14
Necrosis/Ulceration	
No	2
Yes	28
Recurrent after RT	
No	23
Yes	7

### Pre-treatment and treatment evaluation

Diagnosis and staging of disease were based on biopsy followed by CT-scan, MRI, or PET-CT imaging. Full blood counts, glucose levels, biochemical liver and kidney function, and ECG were assessed before recruitment. Thyroid function (TSH, T3, and T4), C-reactive protein (CRP) and creatinine phosphokinase (CPK) levels were also assessed to obtain baseline values for monitoring immunotherapy-related adverse events (irAEs).

Response to cemiplimab and/or IO/RT was documented with direct clinical examination and photography for subsequent analysis, as well as CT/MRI or PET scans if necessary (in cases with bone erosion or nodal/metastatic disease) every 4 months till documentation of complete response (CR), and 6 months thereafter. Clinical assessment of response was performed every 3 weeks on the day of IO administration. We used the RECIST 1.1 criteria to record response [8]**,** with a modification to serve the purpose of the current treatment algorithm. CR refers to elimination of all detectable disease. Partial response (PR) was defined as decrease in the sum of longest diameters (of all irradiated lesions) by more than 30%. Reduction of tumor dimensions by 10–29% was considered as minimal response (MR). Progressive disease (PgD) was documented as an increase of > 20% in the longest dimension. All other cases were recorded as stable disease (SD).

irAEs and RT-related toxicities were recorded by consultation, clinical examination, hematological evaluation, and standard biochemical tests before each cycle of cemiplimab. The serum levels of CPK, CRP, and thyroid function evaluation were recorded every three cycles. The NIH/NCI (National Institute of Health/National Cancer Institute) Common Terminology Criteria for Adverse Events (CTCAE) v 5.0 scale was used to score irAEs and acute radiation toxicity [9]**.**

### IO and RT details

Cemiplimab anti-PD-1 IO was delivered at a dose of 350mg iv., every three weeks. IO was interrupted immediately after development of any grade 2 or higher irAE, excluding controllable thyroid dysfunction.

RT for local disease was given with 12–15-MeV electrons using a linear accelerator (ELEKTA, Precise). The electron energy was defined by measuring the maximum depth of tumors in CTs or MRIs and adding 1cm addition depth to be covered by the 90% isodose curve.

Laterally, the field margins were drawn 2 cm away from the clinical border, taking also into account anatomic constraints. Especially for tumors located on areas overlying brain tissue, special care was taken to avoid overdosage of the brain. As none of the tumors in this study displayed MRI findings of bone involvement, the 90% isodose curve was chosen to cover 0.5 cm beyond the bone surface. This allowed a sharp reduction of the dose to the superficial brain parenchyma, as at 1 cm deeper to the 90% isodose, dose distribution drops to 50% with 12 MeV and 65% with 15-MeV energy. Bolus material of appropriate thickness was used to cover certain thinner areas of the tumor.

Patients received a daily dose of 6 Gy for 6 consecutive daily fractions (physical dose 36 Gy). The estimated biologic dose to normal tissues equivalent to 2 Gy fractionation (EQD2) without time correction was 60 Gy, for α/β = 4Gy. Taking into account the 22-day overall treatment time (OTT) reduction (30 days OTT of a conventionally fractionated scheme delivering 60 Gy vs. 8-day OTT with the hypofractionated schedule), and considering a λ value of 0.2 Gy/day (accounting for normal tissues), the time-corrected biologic dose (EQD2) is estimated to 68Gy [10]. This regimen has been routinely applied in our department, and has been recommended in a meta-analysis showing good cosmetic results [11]**.** The Royal College of Radiologists suggests a 32.5–35 Gy regimen delivered in 4–5 daily fractions for tumors less than 4cm, or 45 Gy in 10 consecutive fractions, with the latter being also supported by the European Society for Radiotherapy and Oncology (ESTRO) for basal cell carcinoma [12, 13]. All the aforementioned regimens provide an EQD2 of approximately 64 Gy, without time correction. The efficacy of hypofractionated RT is similar to conventional [14, 15] and is certainly more convenient, especially for elderly patients, due to the drastic reduction of visits to the RT departments.

### IO/RT algorithm

**Fig. 1:**
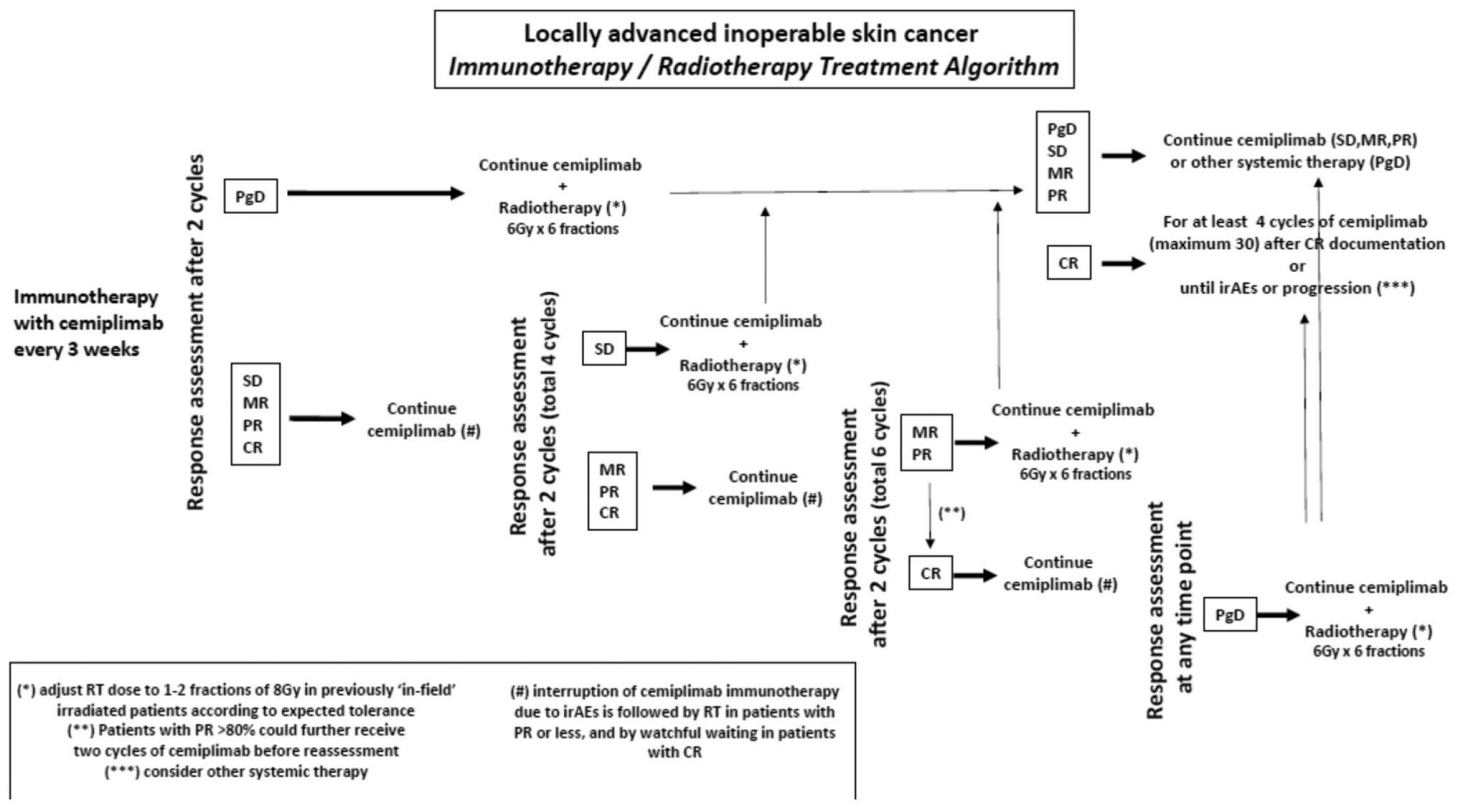
Immuno-radiotherapy treatment algorithm

An algorithmic approach was developed based on complete and incomplete tumor responses to IO and the time-points when RT would be inserted. The treatment algorithm is schematically shown in Fig. 1. Response was recorded on every cycle, and, according to the response, patients underwent RT (non-complete responders) (while continuing cemiplimab) or continued cemiplimab alone (complete responders). For patients who underwent RT, IO continued for at least 4 cycles after achievement of CR. Complete responders after IO, or IO/RT were offered to continue IO for 18 months (till progression or appearance of irAEs). IO interruption due to irAEs was followed by RT in non-complete responders and by watchful waiting in patients who had reached CR. Patients who relapsed after RT were offered palliative systemic therapy, preferably with cetuximab, platinum, and/or antimetabolite combination.

Supplemental Fig. 1s provides a consort flowchart of the study.

### Statistical analysis

The primary endpoint of the study was to build clinical experience on the feasibility and efficacy of IO/RT regimens for LA-sqCSC, and compare the overall response, LPFS and overall and disease-specific survival (OS) rates with those reported in clinical trials of IO. LPFS and OS were defined as the time between start of IO and the first date of documentation of locoregional progressive disease or death from any cause or cancer. LPFS and OS curves were plotted with the Kaplan–Meier method, using the GraphPad Prism version 8.0 statistical package.

## Results

### Immune-related adverse events

Supplemental table 1s reports the irAEs. irAEs enforced interruption of cemiplimab in 6/30 (20.0%) patients (grade 2 skin rash in 3/6, grade 2 kidney toxicity in 2/6, and grade 3 rash/colitis in 1/6). Patients developing irAEs were treated with corticosteroids. Two additional patients (6.6%) developed hypothyroidism (grade I) treated with hormone replacement, while cemiplimab administration was not discontinued.

### Response to upfront IO

**Fig. 2:**
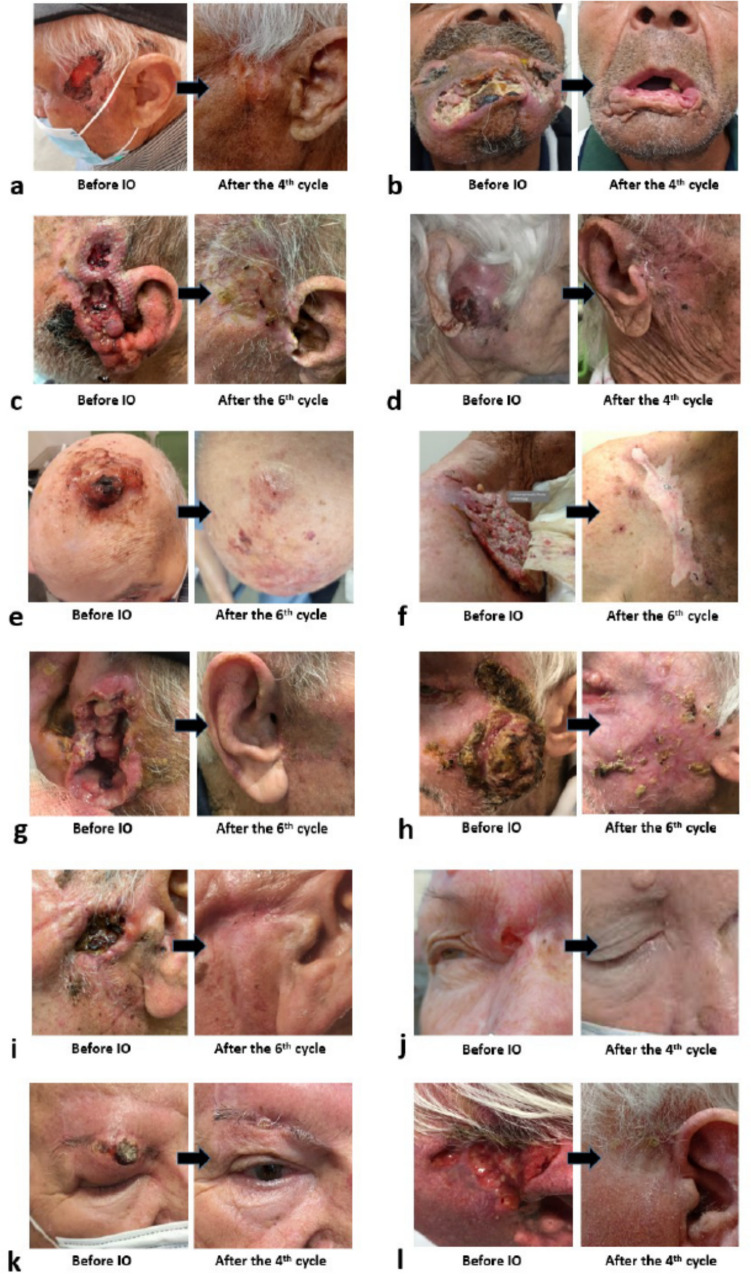
Twelve typical pairs of images of complete response of squamous cell skin carcinomas after 4–6 cycles of immunotherapy (IO) (**a**–**l**)

**Fig. 3:**
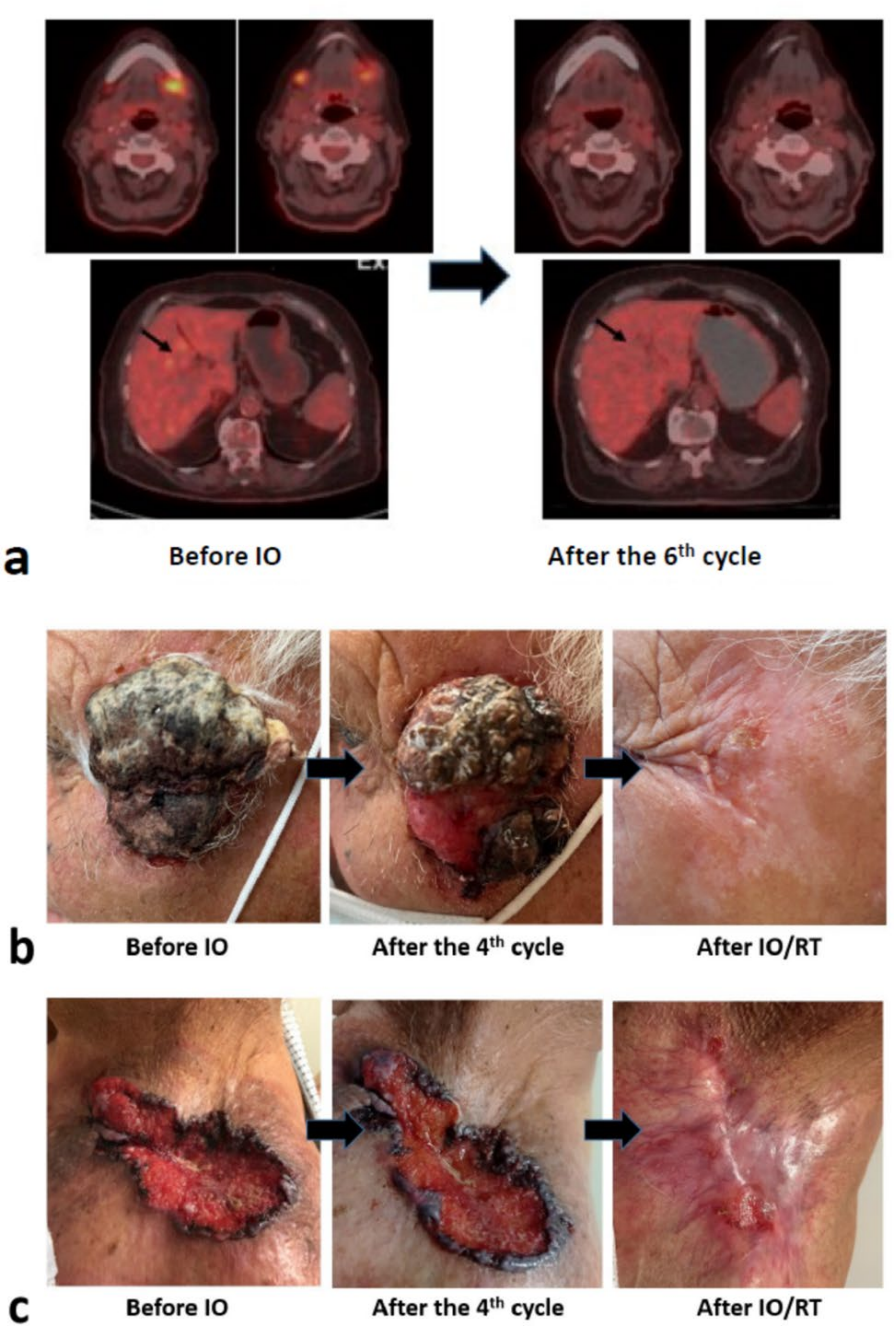
Typical images of complete response of squamous cell skin carcinomas after immunotherapy (IO) or IO/Radiotherapy(RT): **a** a patient with recurrent neck disease and liver metastasis treated with 6 cycles of IO; **b**,**c** patients with persistent disease after 6 cycles of IO that showed complete tumor regression after IO/RT

Table 2 reports the response rates (RR) obtained and the time-points after the onset of IO. After the 4th cycle the RR were 60.0%, with 23.3% of patients having achieved CR. After the 6th cycle the RR were 63.3%, but CRs were recorded in 50.0% of patients. Figures 2 and 3a show photographic documentation of patients who achieved CR.

**Table 2:** Response rates (RR) obtained during immunotherapy (IO) and after radiotherapy (RT)

Response	2nd cycle	4th cycle (*)	6th cycle (**)	After RT	Overall RR
	No pts (%)	No pts (%)	No pts (%)	No pts (%)	No pts (%)
CR	0/30 (0)	7/30 (23.3)	15/30 (50.0)	6/9 (66.7)	21/30 (70.0)
PR	7/30 (23.3)	11/30 (36.7)	4/30 (13.3)	3/9 (33.3)	3/30 (10.0)
MR	10/30 (33.3)	3/30 (10)	8/30 (26.7)	0/9 (0)	1/30 (3.3)
SD	13/30 (43.4)	8/30 (26.7)	2/30 (6.7)	0/9 (0)	4/30 (13.4)
PgD	0/30 (0)	1/30 (3.3)	1/30 (3.3)	0/9 (0)	1/30 (3.3)

CR = complete response, PR = partial response, MR = minimal response, SD = stable disease, PgD = progressive disease, pts = patients

(*) One patient with PgD, one with SD, one with MR, and one with PR refused further IO/RT after the 4th cycle

(**) Three patients with SD refused further IO/RT after the 6th cycle

### Response to IO/RT combination

**Fig. 4:**
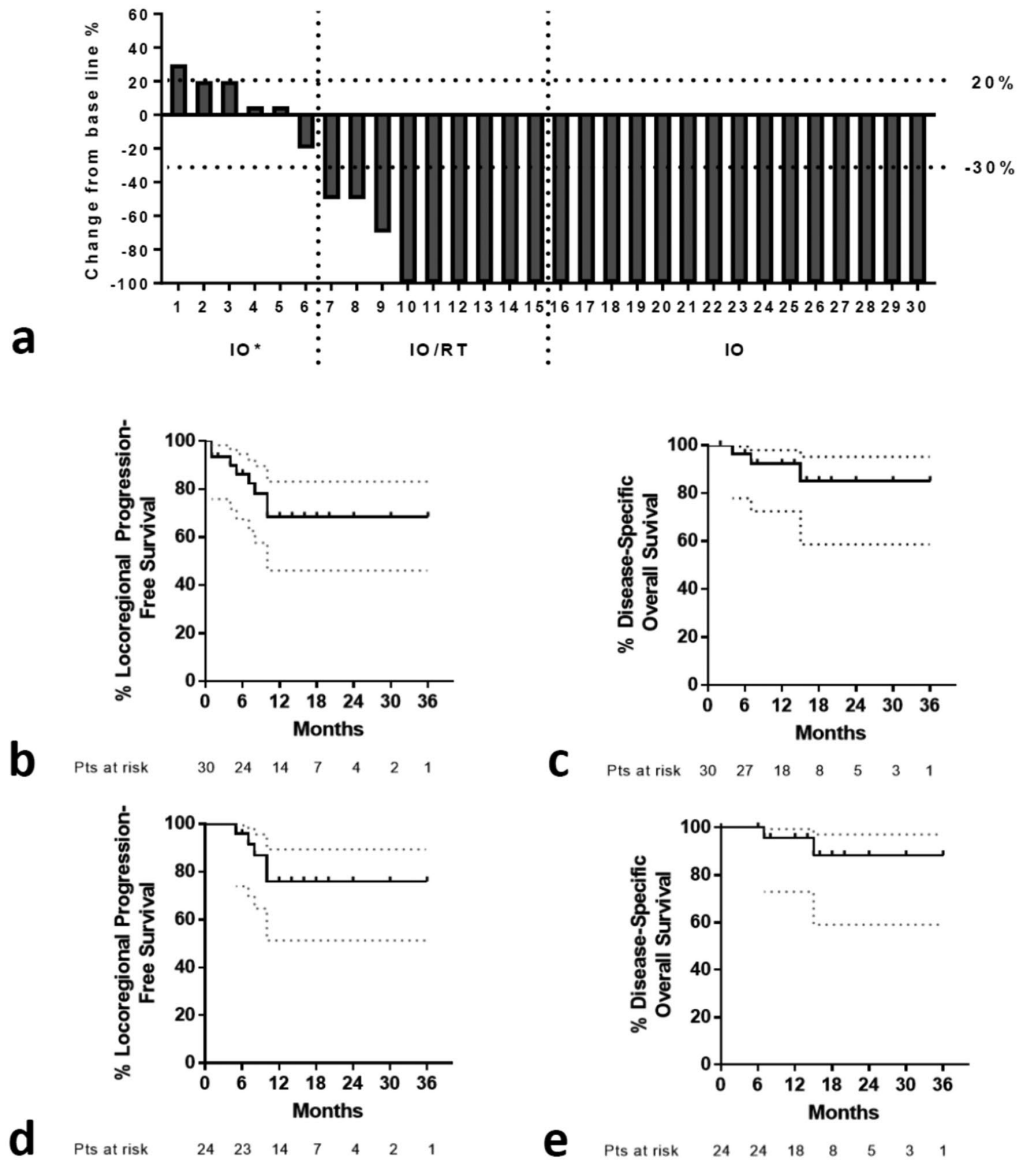
Response and survival: Fig. 4a shows waterfall plot of responses after IO and IO/RT including all 30 patients (IO* refers to patients treated with 6 or less cycles of IO that declined further therapy. None of them had exhibited immune-related adverse events). Figure 4b–d shows Kaplan–Meier locoregional progression-free survival and overall survival. (**b,c** all 30 patients included; **d,e** include 24 patients who complete the prescribed therapy). Dotted lines refer to 95% confidence intervals

Overall, 6 patients (1 patient with PgD, 4 patients with SD, 1 patient with MR) declined further therapy after the 4th–6th cycle (either IO or IO/RT). None of them had exhibited irAEs. Table 2 reports the response rates obtained after RT. As CRs continued IO alone, 9 patients with PR/MR underwent the prescribed hypofractionated RT schedule. Six of them (66.7%) responded completely, and the overall CR rates after IO/RT were 70% (80% overall RR). Figure 3b,c show tumors persistent after 4 cycles of IO that responded completely to RT. Figure 4a shows the waterfall plot of responses after IO and IO/RT, including all 30 patients.

Excluding patients who interrupted cemiplimab due to irAEs or personal reasons, the rest received a median of 12 cycles (range 6–27) of cemiplimab at the time of analysis.

RT early toxicity was minimal, with mild skin erythema (grade 1) noted in all 9 patients. None of the patients have developed late toxicities, including necrosis or skin fibrosis (median follow-up 15 months, range 6–30).

### Survival analysis

Figure 4b,c shows the Kaplan–Meier LPFS and OS (disease-specific) curves, respectively. The 2-year projected LPFS and OS rate were 68.4 and 85.2%, respectively. Overall, 6 patients have passed away at the time of last follow-up, 3 from local progression and 3 from other causes (1 from COVID infection, 1 from heart disease, and 1 from fall). Three patients who had declined further therapy are lost to follow-up.

Analysis of the group of 24 patients who complied to the therapeutic algorithm showed a 2-year projected LPFS and OS rates of 76.0% and 88.2%, respectively (Fig. 4d,e).

## Discussion

Locally far advanced squamous skin carcinomas are often difficult to treat with radical RT for technical or tolerance reasons. Nevertheless, irradiation has been reported to offer around 50% 5-year control rates [16, 17]. Considering the 45–50% CR rates obtained by IO, it is postulated that upfront IO would facilitate the application of RT and eventually, increase the CR and cure rates of this devastating disease. Moreover, patients with CR after IO could enter a watchful waiting policy before administration of RT to recurrence, once this appears.

Beyond the expected additive effect of these two modalities, irradiation of the residual/persistent tumor, would enhance the activity of IO. A large body of experimental evidence suggest that cancer cell irradiation with large fractions triggers the IFN-type-I response, restores HLA-class I molecule expression, and facilitates lymphocyte migration in the tumor [18–20]. All these converge to an increased in situ activation of dendritic and cytotoxic T-cells, enhanced recognition of cancer cells by immune cells, and increased chemo-attraction of activated lymphocytes into the tumor microenvironment. A super-additive result is, therefore, expected, as RT unleashes IO activity and IO eliminates more easily cancer cells that survive the killing effect of RT. This concept is further supported in a study by Nardone et al., where patients with advanced and metastatic sqCSC treated with previous RT exhibited better progression-free survival after cemiplimab immunotherapy [21].

In the current study, we applied a therapeutic algorithm involving upfront cemiplimab, followed by hypofractionated RT in cases with persistent disease after 4 to 6 cycles of IO. The choice of hypofractionated RT was based on both convenience and biology. Since most patients with advanced skin cancer are of advanced age and poor PS, as indeed reported herein, the reduction of the visits to the hospital to a minimum is desirable. Hypofractionated RT is a standard regimen for skin cancer, with equal efficacy to standard RT and good tolerance [11–13, 22, 23]. In addition, as mentioned above, large RT fractions have been postulated to more effectively induce radio-vaccination.

Cemiplimab tolerance was similar to the one recorded in previously published reports [4, 24]**.** Maculopapular rash and fatigue prevailed among side-effects. Despite the small number of 9 cases who were treated with radiation, RT had minimal early toxicity and, for the time being, no fibrosis or necrosis have been documented, suggesting an excellent compatibility between RT and cemiplimab. Concerns have been raised regarding the risk of brain radionecrosis in cases with scalp tumors treated with high-dose electron beam RT [25]**;** De Felice et al. delivered a very high dose of 7–8 fractions of 8 Gy reporting no severe brain toxicity [26]**,** while Ferini et al. suggested a more tolerable scheme of 5 fractions of 7 Gy, which delivers a similar EQD2 to the one prescribed herein [22]. Nevertheless, the methodology applied in our study guaranteed important reduction of dose distribution to the brain parenchyma, resulting in lack of any early or late brain toxicity. In a recently reported study on 102 patients with sqCSC treated with cemiplimab, 22.5% of patients also received RT as induction, consolidation, or palliative regimen, but no case received RT concurrently with cemiplimab [27]**.** Small retrospective studies on sqCSC patients treated with concurrent RT and cemiplimab have confirmed persistent responses with minimal toxicity [28–30].

CR of the disease after the 6th cycle of cemiplimab was noted in 50.0% of patients, similar to the one reported in published clinical trials [27, 31]**.** This increased to 70% after the addition of RT. Beyond the efficacy of hypofractionated RT, a super-additive synergy can be postulated, as a ‘radio-vaccination’ effect could have enhanced the activity of cemiplimab. Moreover, as RT is given by electrons, with minimal exposure of the bone marrow, lymph nodes and circulating lymphocytes, lymphopenia was undetectable (data not shown). The immunosuppressive effects of RT have been postulated to be the cause of the disappointing results of IO/RT combination in squamous cell head-neck cancer [32]**,** for indeed RT has a strong lymphopenia-inducing effect on these patients [33]**.** In the negative head-neck IO/RT trials, prolonged treatment therapy and irradiation of the neck vessels and lymph nodes had been applied, in contrast to the current study [34, 35]**.**

Limitations of the study include the low number of patients who received RT; thus, underestimation of radiation toxicities and uncertainties on the eradication rates reported cannot be excluded. The 20% rate of patients who declined further therapy after the 4th–6th cycle is also a weakness of the study, although this was a result related to the poor PS and advanced age of patients.

It is concluded that upfront IO with cemiplimab followed by early onset of RT in patients with incomplete response after 4 to 6 cycles of IO, appears as a promising therapy in patients with locally advanced inoperable and difficult to treat radically with RT squamous cell skin cancer.

## Supplementary Information

Below is the link to the electronic supplementary material.

Supplementary file1 (DOCX 71 kb)

## Data Availability

No datasets were generated or analyzed during the current study.
