# Supplementary material for: Integration of immunotherapy and radiotherapy in a therapeutic algorithm for locally advanced squamous cell skin cancer
Source: Med Oncol. 2025 Jun 4;42(7):238. doi: 10.1007/s12032-025-02785-3 (PMC12137472; doi:10.1007/s12032-025-02785-3)
Supplement: Supplementary file 1 — Supplementary file1 (DOCX 71 kb) [file 12032_2025_2785_MOESM1_ESM.docx]

**Supplemental Figure 1S**

Consort Diagram.


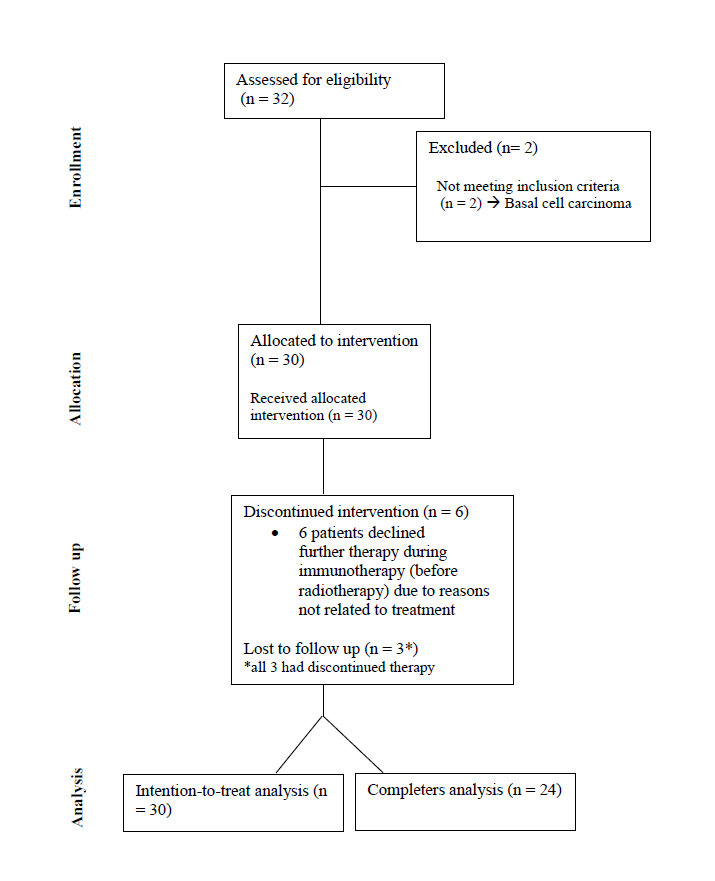


**Supplemental Table 1S**

Immune-related adverse events (irAEs) noted in 30 patients, time of onset (expressed as the number of last cemiplimab cycle), and immunotherapy (IO) discontinuation.

| **irAEs** | **No pts** | **% of total pts** | **IO-cycle** | **IO discontinuation** |
| --- | --- | --- | --- | --- |
| Maculopapular rash (**) | 3 | 10 | 9, 10, 11 | yes |
| Rash/colitis (***) | 1 | 3.3 | 16 | yes |
| Acute kidney injury (**) | 2 | 6.6 | 4, 14 | yes |
| Hypothyroidism (*) | 2 | 6.6 | 12, 17 | no |

pts=patients

(*) grade 1, (**) grade 2, (***) grade 3
